# Color Constancy *via* Multi-Scale Region-Weighed Network Guided by Semantics

**DOI:** 10.3389/fnbot.2022.841426

**Published:** 2022-04-08

**Authors:** Fei Wang, Wei Wang, Dan Wu, Guowang Gao

**Affiliations:** ^1^School of Electronic Engineering, Xi'an Shiyou University, Xi'an, China; ^2^State Key Laboratory of Advanced Design and Manufacturing for Vehicle Body, Hunan University, Changsha, China; ^3^School of Telecommunications Engineering, Xidian University, Xi'an, China

**Keywords:** color constancy, multi-scale, weight pooling layer, semantic, network

## Abstract

In obtaining color constancy, estimating the illumination of a scene is the most important task. However, due to unknown light sources and the influence of the external imaging environment, the estimated illumination is prone to color ambiguity. In this article, a learning-based multi-scale region-weighed network guided by semantic features is proposed to estimate the illuminated color of the light source in a scene. Cued by the human brain's processing of color constancy, we use image semantics and scale information to guide the process of illumination estimation. First, we put the image and its semantics into the network, and then obtain the region weights of the image at different scales. After that, through a special weight-pooling layer (WPL), the illumination on each scale is estimated. The final illumination is calculated by weighting each scale. The results of extensive experiments on Color Checker and NUS 8-Camera datasets show that the proposed approach is superior to the current state-of-the-art methods in both efficiency and effectiveness.

## 1. Introduction

The observed color of an object in an image (representing the observed values in RGB space) depends on the intrinsic color and light-source color. It is quite easy to distinguish the reflectance from the light-source color for human beings while endowing a computer with the same ability is difficult (Gilchrist, 2006). For example, given a red object, how can one discern if it is a white object under red light or a red object under a white light? To assist a computer in solving this problem, it is necessary to separate the color of the light source, namely, the color constancy.^1^ The goal of computational color constancy is to preserve the perceptive colors of objects under different lighting conditions by removing the effect of color casts caused by the scene's illumination.

Color constancy is a fundamental research topic in the image-processing and computer-vision fields, and it has many applications in photographic technology, object recognition, object detection, image segmentation, and other version systems. Color casts caused by incorrectly applied computational color constancy can negatively impact the performance of image segmentation and classification (Afifi and Brown, 2019; Xue et al., 2021), thus, there is a rich body of work on this topic. Generally, methods for obtaining color constancy with image data are divided into two main categories: low-level-feature-based methods (Buchsbaum, 1980; Brainard and Wandell, 1986; Lee, 1986; Wandell and Tominaga, 1989; Nieves et al., 2000; Krasilnikov et al., 2002; Weijer et al., 2007; Gehler et al., 2008; Tan et al., 2008; Toro, 2008; Gijsenij et al., 2011; Finlayson, 2013; Gao et al., 2013; Barron, 2015; Bianco et al., 2015, 2017; Cheng et al., 2015; Shi et al., 2016; Xiao et al., 2020; Yu et al., 2020) and semantic-feature-based methods (Schroeder and Moser, 2001; Spitzer and Semo, 2002; Van De Weijer et al., 2007; Bianco et al., 2008; Lau, 2008; Li et al., 2008; Gao et al., 2015; Afifi, 2018).

*Low-level-features-based methods* pay attention to the law of the color of the image itself, and they do not consider the image-content information. These methods consider the relationship between color and achromatic color statistics (Weijer et al., 2007; Gehler et al., 2008), inspired by the human visual system (Nieves et al., 2000; Krasilnikov et al., 2002; Gao et al., 2013), spatial derivatives, and frequency information of scene illuminations on the image (Nayar et al., 2007; Joze and Drew, 2014), extract hand-crafted features from training data (Buchsbaum, 1980; Brainard and Wandell, 1986; Finlayson, 2013; Cheng et al., 2015), and learn features automatically by a convolutional neural network (CNN) from samples (Barron, 2015; Bianco et al., 2015, 2017; Shi et al., 2016; Xiao et al., 2020; Yu et al., 2020). Although these methods have achieved good results, especially the CNN-based methods, various methods are used to make the illumination estimation as accurate as possible, but in some complex situations, due to inflexibility, they cannot well solve the color ambiguity.

*Semantic-feature-based methods* are more in line with human vision. When observing a scene, human beings have a certain psychological memory of the color of the object itself in the scene. Therefore, the content of the scene can play a certain guiding role in color constancy. Because previous attempts at semantic information extraction have not been accurate, there is relatively little research on this type of algorithm. Van De Weijer et al. (2007) proposed a color-constancy algorithm based on advanced visual information. The algorithm models the image into many semantic categories, such as sky, grassland, road, pedestrian, and vehicle, and it calculates multiple possible illumination values from these semantic categories. Each illumination is used to correct the image and calculate the semantic combination with the greatest probability. At this time, the illumination is the optimal scene illumination. Schroeder and Moser (2001) divided images into different categories, and then they learned different features for each type. Bianco et al. (2008) proposed an indoor and outdoor adaptive illumination estimation algorithm that uses a classification algorithm to divide the image into indoor and outdoor scenes, and then they estimated the illumination according to the parameters learned by training data. Afifi (2018) exploited the semantic information together with the color and spatial information of the input image, and they trained a CNN to estimate the illuminant color and gamma correction parameters. This is one of the most effective methods of this type.

However, these two methods may not find the optimal solution in some complex situations due to inflexibility. To summarize, several open problems remain unsolved in these approaches, which can be generally concluded to have two aspects.

- *Color ambiguity with only low-level features:* Many of these methods (Buchsbaum, 1980; Brainard and Wandell, 1986; Lee, 1986; Wandell and Tominaga, 1989; Nieves et al., 2000; Krasilnikov et al., 2002; Weijer et al., 2007; Gehler et al., 2008; Tan et al., 2008; Toro, 2008; Gijsenij et al., 2011; Finlayson, 2013; Gao et al., 2013; Cheng et al., 2015) only focus on the color of the image itself, for images with large color deviation, it is difficult to accurately estimate the illumination. The development of CNNs has facilitated a qualitative leap in illumination estimation, but many CNN-based methods (Barron, 2015; Shi et al., 2016; Bianco et al., 2017) are patches-based, which take the small sampled image patches as input and learn the corresponding local estimations subsequently pooled into a global result. The small patches contain less contextual information, which commonly leads to ambiguity in local estimation. When inferring the illumination color in a patch, it is often the case that the patch contains little or no semantic context benefiting its reflectance or illumination estimation. To solve this problem, Hu et al. (2017) used a global image as input, and they designed a confidence weight layer to learn the weight of each patch. Afifi and Brown (2020) proposed an end-to-end approach to learn the correct white balance, which consists of a single encoder and multiple decoders, mapping an input image to two additional white-balance settings corresponding to indoor and outdoor illuminations. Both methods achieved great success. However, due to the lack of attention to semantic information, large errors in illumination estimation exist in some scenes, which has also been found in our experiments.
- *Inaccurate illumination with only semantics:* Owing to the low accuracy of semantic segmentation, the early semantic-based color-constancy algorithm has great limitations. CNNs have greatly improved the accuracy of semantic segmentation. Afifi (2018) exploited the semantic information together with the color and spatial information of the input image, and they trained a CNN to estimate the illuminant color and gamma correction parameters. However, there is a possibility of error in segmentation, and incorrect segmentation will lead to errors in illumination estimation. In our experiments, we also verified that incorrect segmentation can lead to incorrect illumination estimates.

To address the aforementioned open problems, we did some experiments. In one experiment, we conducted, yellow banana and a red apple were placed into a scene, illuminated with different colors of light, and then different observers were allowed to view the results. It was found that the observers can correctly distinguish the color of the fruit because humans generally think that bananas are yellow and apples are red. It can be seen that objects with inherent colors can guide observers to estimate the lighting of the scene; i.e., the objects in the scene have a great effect on the color constancy of human vision. In addition, we conducted an experiment in which some objects were placed in the scene to allow the observer to observe them from different distances. It was found that the colors of certain areas in the scene observed at different distances were biased (this deviation is relatively small), but the bias did not have much impact on the overall color. In addition, in the present study, traditional algorithms were used to estimate the illumination of the image at different scales. It was found that the estimated illumination at different scales exhibits some deviations, but they were all close to the actual illumination. It can be thought that scale has a certain influence on the color constancy of human vision.

Estimating multiple illuminations from one image at multiple scales is also in line with a verification conclusion obtained in Shi et al. (2016), namely, that multiple hypothetical illuminations from one image can help improve the accuracy of illumination estimation. Inspired by that, we propose herein a learning-based **M**ulti-scale **R**egion-**w**eighed **N**etwork guided by **S**emantics (MSRWNS) to estimate the illuminated color of the light source in a scene. First, the semantic context of an image is extracted, the image and its semantics are put into the network, and through a series of convolution layers, the region weights of the image at different scales are obtained. Then, through the weight-pooling layer (WPL), the illumination estimation on each scale is obtained. The global illumination is calculated by weighting on each scale.

The MSRWNS network differs from the existing methods and has three contributions, which follow.

- It estimates multiple global illuminations at different feature scales, and it obtains the final lighting by simple weighting.
- Different from previous semantic-based methods, while using semantic guidance, a new region-WPL is used. The network layer simultaneously learns the contribution and local illumination of different regions in the image at each scale. It can, thus, effectively solve the illumination estimation error caused by the semantic segmentation error.
- A large strip is used in the convolution to replace the max pooling layer in the network, which improves the speed of light estimation without reducing accuracy.

The rest of this article is organized as follows. In section 2, the structure of the proposed network and training strategy is presented, together with the related experimental content in section 3. Conclusions are given in section 4.

## 2. Multi-Scale Region-Weighed Network Guided by Semantics

Following the widely accepted simplified diagonal model (Finlayson et al., 1994; Funt and Lewis, 2000), the color of the light source is represented as


(1) $$\begin{array}{l} I_{c}=E_{c}\times R_{c},c\in \left\{r,g,b\right\}, \end{array}$$

where *I*_*c*_ = {*I*_*r*_, *I*_*g*_, *I*_*b*_} is the color image under an unknown light source, *R*_*c*_ = {*R*_*r*_, *R*_*g*_, *R*_*b*_} the color image recorded by a white-light source, and *E*_*c*_ = {*E*_*r*_, *E*_*g*_, *E*_*b*_} the light source needed to be estimated from *I*_*c*_.

A new color-space model has been used by color-constancy methods (Finlayson et al., 2004; Barron, 2015; Shi et al., 2016) in recent years and has certain advantages, i.e., *log* − *uv* space. ^2^ The calculation method proceeds as follows:


(2) $$\begin{array}{l} L_{u}=log\left(R/G\right),L_{v}=log\left(B/G\right). \end{array}$$

After estimating the light, it can be converted back to RGB space through a very simple formula:


(3) $$\begin{array}{l} \begin{array}{c} R=exp\left(-L_{u}\right)/z,G=1/z,B=exp\left(-L_{v}\right)/z\\ z=\sqrt{\exp{\left(-L_{u}\right)}^{2}+\exp{\left(-L_{v}\right)}^{2}+1}, \end{array} \end{array}$$

where (*L*_*u*_, *L*_*v*_) is the image in *log* − *uv* color space. (*R, G, B*) is the image in RGB color space.

### 2.1. Problem Formulation

Generally, we only know the image *I*_*c*_ under an unknown light source *E*_*c*_ that must be estimated. The goal of color constancy is to estimate *E*_*c*_ from *I*_*c*_ and then compute it as *E*_*c*_ = *I*_*c*_/*R*_*c*_. How do we estimate *E*_*c*_ from *I*_*c*_? To address this problem, we formulate color constancy as a regression problem.

First, we obtain the semantic context of the image, and for this, we use PSPNet (Zhao et al., 2016), defined as *I*_*s*_, and then convert *I*_*c*_ from RGB space to *log* − *uv* space to obtain (*I*_*u*_, *I*_*v*_). Combining these three channels into a new three-channel image *I*_*n*_, the aim is to find a mapping *f*_θ_ such that *f*_θ_(*I*_*n*_) = *P*_*uv*_, where *P*_*uv*_ represents the light value in *log* − *uv* space.

As mentioned earlier, the objects in the scene have a great effect on the color constancy of human vision (Van De Weijer et al., 2007; Gao et al., 2019). Therefore, the designed color-constancy algorithm should imitate the human visual system, i.e., the mapping *f*_θ_ should be able to be based on semantic information and is used to support the larger contribution area and suppress the smaller contribution area in the image. Therefore, two aspects must be considered in the model: First, one must find a way to estimate the illumination of each area in the image, and, second, one must use an adaptive algorithm to integrate the illumination of these multiple areas into a global illumination. Supposing that *R* = *R*_1_, *R*_2_, ..., *R*_*n*_ represents *n* non-overlapping regions in the image *I*_*c*_, ${E_{uv}}^{i}$ represents the estimated scene illumination of the *i*th area *R*_*i*_. Therefore, the mapping *f*_θ_ can be expressed as follows:


(4) $$\begin{array}{l} f_{\theta }\left(I_{n}\right)=P_{uv}=\overset{n\text{- 1}}{\underset{i=\text{0}}{\sum }}w\left(R_{i}\right)E_{uv}^{i}, \end{array}$$

where *w*(*R*_*i*_) represents the contribution of each area to the illumination estimation, i.e., the weight. In other words, if *R*_*i*_ contains the semantic context information, the corresponding *w*(*R*_*i*_) has a higher weight value.

### 2.2. Network Architecture

**Figure 1:**
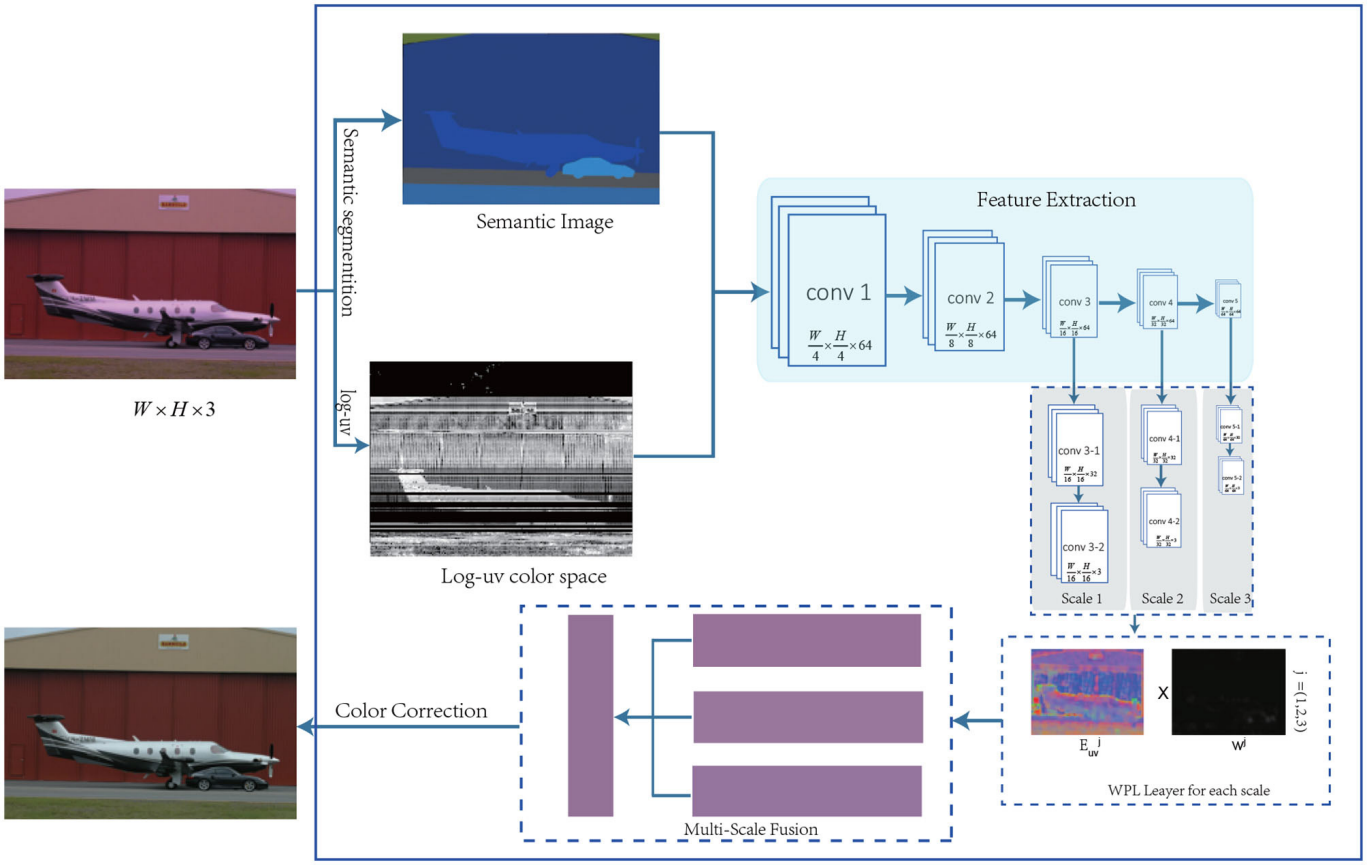
The architecture of Multi-scale Region-weighed Network guided by Semantics (MSRWNS) is trained to estimate the illuminant and weights of a given image in each region.

It can be seen from Equation (4) that it is necessary to design a network structure *f*_θ_ to be able to calculate *w*(*R*_*i*_) and $E_{uv}^{i}$ in each area. The network structure is shown in Figure 1.

We learn *P*_*uv*_ at different scales from the intermediate features with scales of 1/16, 1/32, and 1/64. Defining the superscript *j* to represent the scale, ${P_{uv}}^{j}$ then represents the estimated illumination in *log* − *uv* space under the *j*th scale, which is converted back to RGB space according to Equation (3) to obtain ${P_{c}}^{j}$, where *c* = *R, G, B*. Finally, the final illumination *P*_*c*_ in RGB color space is obtained by simple calculation of the obtained illumination on different scales, and the formula is:


(5) $$\begin{array}{l} \begin{array}{c} P_{c}=\overset{n}{\underset{j=1}{\sum }}C_{j}P_{\text{c}}^{\text{j}}\\ \overset{n}{\underset{j=1}{\sum }}C_{j}=1, \end{array} \end{array}$$

where *C*_*j*_ represents the weight of the illumination obtained at each scale. In this article, *j* = 1, 2, 3. In the final illumination calculation, it is assumed that the estimated illumination at different scales contributes the same to the scene illumination, namely, *C*_*j*_ = 1/3, *j* = 1, 2, 3.

### 2.3. Weight-Pooling Layer

In most of the previous methods, the extracted features are directly calculated through several fully connected layers to obtain a global illumination. However, it can be seen from the results of the earlier literature that the effect of illumination estimation is not significantly improved. Referring to Hu et al. (2017), we used a custom network layer, called a WPL, the main function of which is to converge the regional illumination into a global illumination, and at the same time, learn the weight *w*(*R*_*i*_) of each area; *R*_*i*_ represents the *i*th area. The *WPL* on each scale is expressed as follows:


(6) $$\begin{array}{l} P^{j}=\overset{n\text{- 1}}{\underset{i=\text{0}}{\sum }}{w_{i}}^{j}{E_{i}}^{j}, \end{array}$$

where *P*^*j*^ represents the output of the WPL layer in the *j*th scale, ${w_{i}}^{j}$ the contribution of each region that must be learned on the *j*th scale, ${E_{i}}^{j}$ the illumination of the *i* area on the *j*th scale that must be learned, and *n* represents the number of areas. In this study, each scale is $n=\frac{W}{16}\times \frac{H}{16},\frac{W}{32}\times \frac{H}{32},\frac{W}{64}\times \frac{H}{64}$.

### 2.4. Illumination Fusion for Multiple Scales

We made a simple attempt to determine how to select illumination at multiple scales, and we used several samples to train the 3 classification problems, hoping to obtain the probability of illumination at different scales. However, the training process model is difficult to converge and the effect is not ideal. Finally, for the sake of simplicity, it is assumed that each scale has the same contribution to the illumination estimation, so the average value of 3-scale illumination is taken as the final illumination in this section^3^, and the results also show that the average value is higher than that of a single scale on a variety of datasets.

### 2.5. Loss Function

At the time of training optimization, Euclidian loss is utilized for the network, defined


(7) $$\begin{array}{l} Loss=\overset{n}{\underset{j=1}{\sum }}Loss_{j}, \end{array}$$

(8) $$\begin{array}{l} Loss_{j}=\frac{1}{N}\overset{n}{\underset{i=1}{\sum }}{\left\|E_{i}^{e}-E_{i}^{t}\right\|}^{2}, \end{array}$$

where *Loss*_*j*_ is the loss function of the *j*th scale, *E*^*e*^ is the illumination estimated by the network, *E*^*t*^ is the ground truth illumination, and *N* represents the batch of the training samples. The loss is minimized using stochastic gradient descent with standard back-propagation.

### 2.6. Discussion of Network Structure

Either shallower (i.e., Shi et al. 2016) or deeper networks (i.e., VGG-net Simonyan and Zisserman 2014) could replace the pre-feature extraction in the proposed system. However, due to the color-constancy problem, the best network for feature extraction should have enough capacity to distinguish ambiguities and should be sensitive to different illuminants. We tried several common networks, such as AlexNet (Krizhevsky et al., 2012), VggNet16 (Simonyan and Zisserman, 2014), and VggNet19 (Simonyan and Zisserman, 2014), and they all achieved good results. Finally, to improve the computational efficiency of the network, we simplified AlexNet (Krizhevsky et al., 2012) and removed all of its pooling layers. The test results showed that the efficiency increased by approximately 4 and the accuracy by an average of 5.2%.

## 3. Experimental Results

### 3.1. Datasets

Because semantic information is needed in this study, we mainly used a semantic segmentation dataset, namely, the ADE20k dataset (Zhou et al., 2016). Meanwhile, we used PSPNET (Zhao et al., 2016; Zhou et al., 2017)^4^ to segment the semantic information for Color Checker (Gehler et al., 2008; Zhou et al., 2017) and NUS 8-Camera datasets (Cheng et al., 2014).

During the training process, 100 images with accurate semantic segmentation were manually selected from the Color Checker dataset (Gehler et al., 2008), and 200 images were extracted from the NUS 8-Camera dataset (Cheng et al., 2014).

In addition, since the ADE20k dataset (Zhou et al., 2017) does not provide illumination information, it is assumed that the images in the ADE20k dataset are corrected white-balanced images, and we, therefore, visually selected 500 images with normal color. Different lights were then rendered according to the following equations:


(9) $$\begin{array}{l} {I_{i}}^{\prime }=I_{i}.M_{i}, \end{array}$$

(10) $$\begin{array}{l} M_{i}=\left[\begin{array}{ccc} r_{i} & 0 & 0\\ 0 & g_{i} & 0\\ 0 & 0 & b_{i} \end{array}\right], \end{array}$$

where (*r*_*i*_, *g*_*i*_, *b*_*i*_) represents the simulated scene illumination. After simulation, more than 2,000 training images with illumination labels and accurate semantic information were obtained from the ADE20k (Zhou et al., 2017) dataset. These 2,000 images were cut, mirrored horizontally and vertically, and rotated from (−30^*o*^, 30^*o*^), 90^*o*^, 180^*o*^, and a total of approximately 20,000 pieces of data were obtained. Similarly, the images selected from the Color Checker (Gehler et al., 2008) and NUS 8-Camera datasets (Cheng et al., 2014) were processed in the same way to obtain approximately 8,000 images. All 28,000 images were randomly cropped and normalized to 512 × 512 as network input. As in the previous study, 3-fold cross-validation was used for all of the datasets, and each one run was used for training, one for validation, and one for testing.

### 3.2. Metrics

Color-constancy algorithms are often evaluated using a distance measure, such as Euclidean distance (Land, 1977; Buchsbaum, 1980), perceptual distances (Gijsenij et al., 2009), reproduction angular error (Finlayson et al., 2016), and angular error (Hordley and Finlayson, 2004; Cheng et al., 2014; Bianco et al., 2015; Shi et al., 2016). Within these metrics, the angular error is the most widely used in this field, and most of the existing works (Cheng et al., 2014; Bianco et al., 2015; Shi et al., 2016) that tested using the Color Checker (Gehler et al., 2008), NUS 8-Camera (Cheng et al., 2014), and ADE20k datasetd (Zhou et al., 2017) reported their performance in terms of the angular error in five indexes: *Mean, Median*, and *TriMean* of all errors; mean of the lowest 25% of errors (*Best* 25%); and mean of the highest 25% of errors (*Worst* 25%). Hence, in the present study, we also used these indexes. In particular, although we aimed to estimate the single illuminant in this study, we also tested performance on the popular outdoor multi-illuminant dataset (Arjan et al., 2012). The angular error between the estimated illuminant *E*_*e*_ and ground-truth illuminant $E_{e}^{*}$ is computed for each image as follows:


(11) $$\begin{array}{l} e=\arccos\left(\frac{E_{e}.E_{e}^{*}}{\left\|E_{e}\|.\|E_{e}^{*}\right\|}\right). \end{array}$$

The less the value of *e* is the better performance of the method.

### 3.3. Implementation Parameters

In this subsection, the parameter settings for training our final model are given.

**Figure 2:**
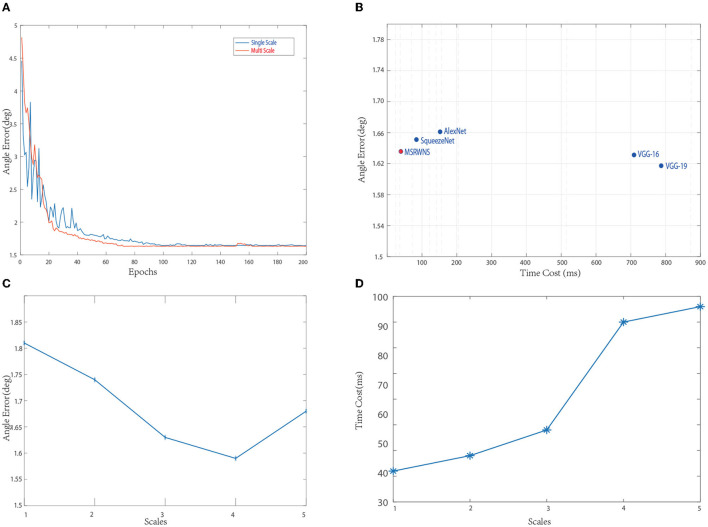
**(A–D)** Performance under different parameters.

**Feature-extraction-network selection:** Different network structures, such as AlexNet (Krizhevsky et al., 2012), VGGNet-19 (Simonyan and Zisserman, 2014), and SqueezeNet (Iandola et al., 2016), were used to test performance. The comparison diagram is shown in Figure 2B, from which it can be seen that, although VGGNet-16 (Simonyan and Zisserman, 2014) and VGGNet-19 (Simonyan and Zisserman, 2014) network structures have a better effect than other networks, they take more time. Finally, considering effect and efficiency, the structure in Figure 1 is used in this study.

**Network input and output:** We compared the performance of the model trained with (*I*_*u*_, *I*_*v*_, *I*_*t*_) three-channel image input and (*I*_*u*_, *I*_*v*_) two-channel input, and we tested the performance of different resolution images as network input. The comparison results are shown in Table 1. Considering effect and efficiency, the effect is the best when the network input image resolution is 512 and semantic information is used at the same time.

**Table 1:** Accuracy of different network input sizes and whether semantics are used or not in the Color Checker dataset.

**Size/Method**	**Mean**	**Median**	**TriMean**	**Best 25%**	**Worst 25%**	**Speed(ms)**
*256,T*	1.67	1.36	1.53	0.45	4.09	27
*256,None*	1.81	1.44	1.62	0.55	4.31	23
*512,T*	1.64	1.17	1.28	0.31	3.82	34
*512,None*	1.66	1.33	1.48	0.42	4.01	30
*128,T*	1.74	1.33	1.49	0.55	4.17	19
*128,None*	1.76	1.35	1.51	0.55	4.27	15

*256, 512, and 128 are defined as different input sizes. T, None defined as the use of semantics or not. Red indicates best accuracy*.

**Figure 3:**
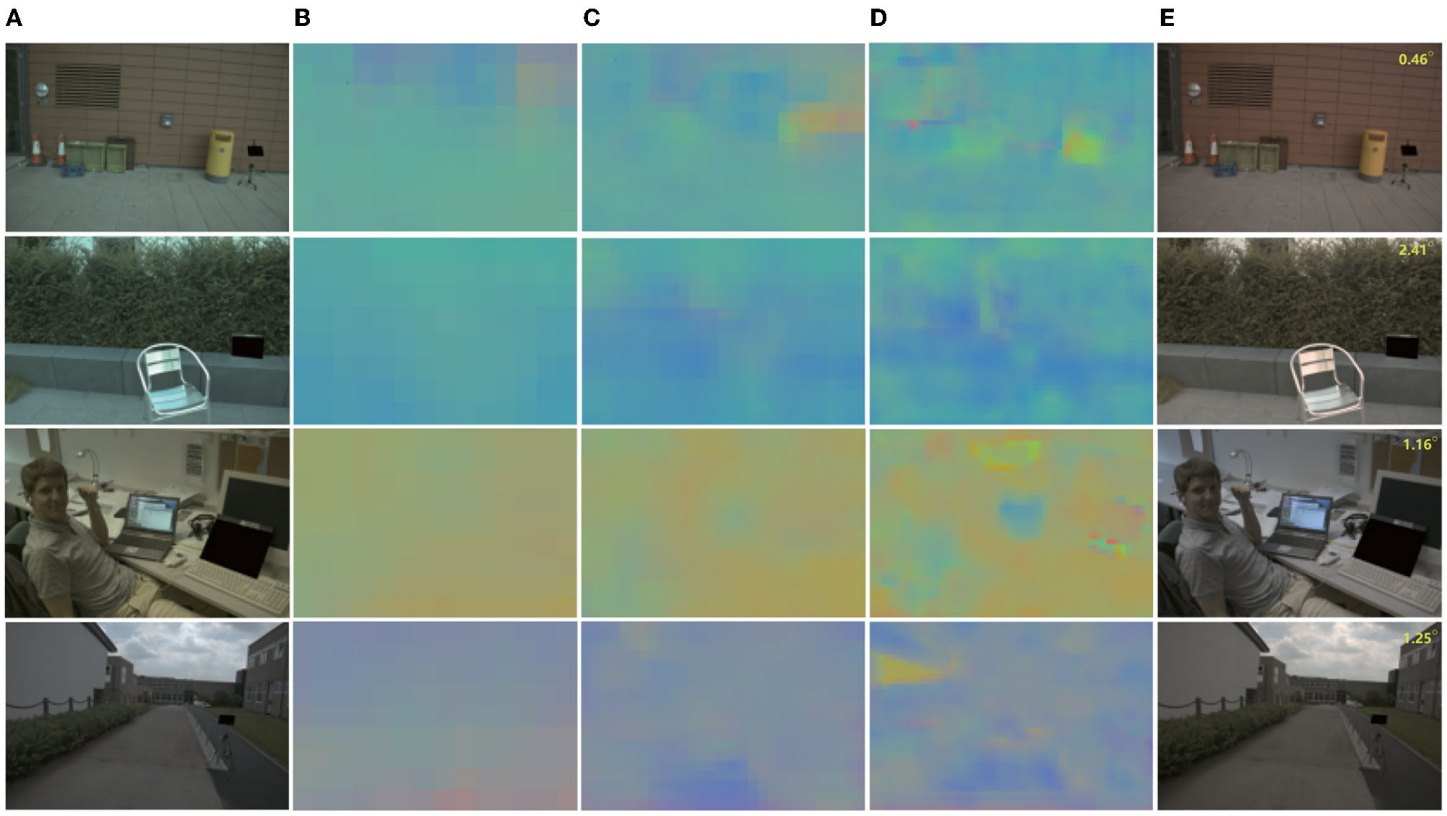
Local illumination estimation of network output at different scales. Left to right: **(A)** original color-biased image; **(B)** Local illumination at scale 3; **(C)** Local illumination at scale 2; **(D)** Local illumination at scale 1; **(E)** Final correction results. It can be seen from the figure that under different scales, due to different area sizes, the estimated illumination is also different, but the overall color is basically the same.

For output, we tested the effects of 1–5 scales outputs on illumination estimation, where 1 scale uses $\frac{W}{64}\times \frac{H}{64}$, 2 scales uses $\frac{W}{32}\times \frac{H}{32},\frac{W}{64}\times \frac{H}{64}$, 3 scales use $\frac{W}{16}\times \frac{H}{16},\frac{W}{32}\times \frac{H}{32},\frac{W}{64}\times \frac{H}{64}$, 4 scales use $\frac{W}{8}\times \frac{H}{8},\frac{W}{16}\times \frac{H}{16},\frac{W}{32}\times \frac{H}{32},\frac{W}{64}\times \frac{H}{64}$, and 5 scales use $\frac{W}{8}\times \frac{H}{8},\frac{W}{16}\times \frac{H}{16},\frac{W}{32}\times \frac{H}{32},\frac{W}{64}\times \frac{H}{64},\frac{W}{128}\times \frac{H}{128}$. The curves are shown in Figures 2C,D. It can be seen that the effect is best when the scale is 4, but the time consumption is more than doubled when the scale is 3. Considering comprehensive effect and efficiency, we used 3 scales in the network. Figure 3 shows the intermediate results of estimating illumination at different 3 scales.

**Figure 4:**
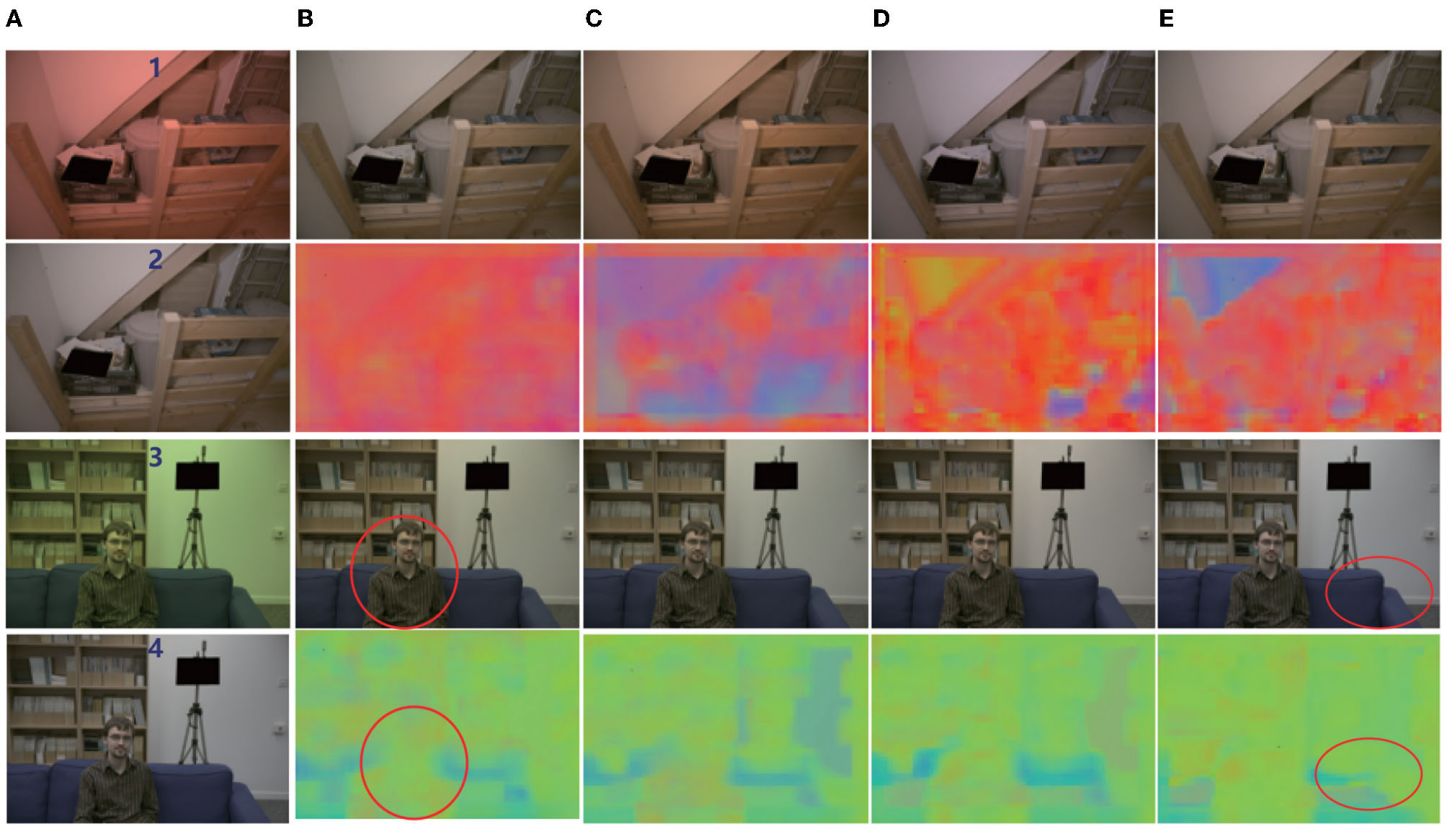
Effect of training at different epochs. Left to right: **(A)** original image and normal image; **(B)** output image and local illumination at 100 epochs; **(C)** output image and local illumination at 500 epochs; **(D)** output image and local illumination at 2,000 epochs; **(E)** output image and local illumination 4,000 epochs. It can be seen that with an increasing number of epochs, the corrected image tends to the real result. In addition, it can be seen from the red circle that the area in which people are located and that at the junction of the wall and sofa are different in local color.

**Batch size and learning:** For optimization, Adam (Kingma and Ba, 2014) was employed with a batch size of 64, and a basic learning rate of 0.0001 was set for training. We trained all of the experiments over 4,000 epochs (2,50,000 iterations with batch size 64). The average angular error is calculated in the Color Checker dataset for every 20 epochs. The curve is shown in Figure 2A. Figure 4 shows the resulting image and local illumination after different numbers of epochs.

### 3.4. Comparison With State-of-the-Art Methods

**Figure 5:**
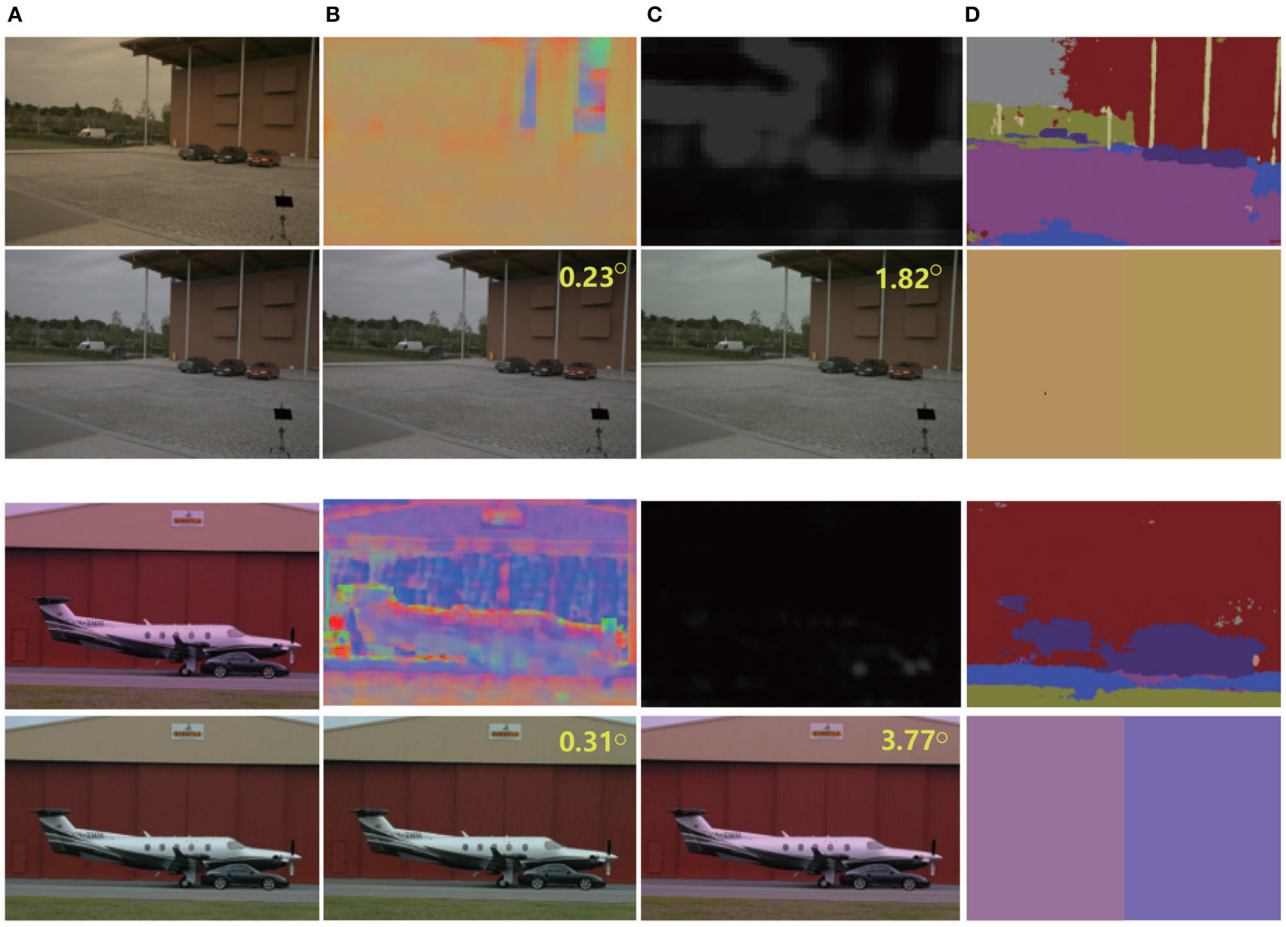
Effect comparison between using weight-pooling layer (WPL) layer or not using it. For each group, **(A)** original image and normal image; **(B)** local illumination and image with WPL layer; **(C)** weight image and result without WPL; **(D)** semantic and global illumination (in global illumination, the left-hand side is the light estimated after using WPL and the right-hand side is that estimated without WPL). It can be seen that in an image with rich scenic content, the effect of using the WPL layer is significantly better than that of not using it. From the weighted image (the image after the weight is normalized, for which the gray value is high, the weight is large, and vice versa) and the local illumination image, different objects in the scene have different weights, and some contribute significantly. It can be seen from the estimated illumination and semantics that the illumination is also different in different semantic parts, and the approximate shape of the objects in the scene can be seen from the illumination image.

To evaluate the performance of the proposed method and the influence of the WPL layer on the method. We trained two models. One used the mean value when the local region converges to the global, which is defined as *MSRWNS-AVG*, the other model used the *WPL* layer, which is defined as *MSRWNS*. In addition, we also estimated the illumination effect at each scale, defined as *MSRWNS-1, MSRWNS-2*, and *MSRWNS-3*. Several visualizations of processing outputs obtained using the proposed method are presented in Figure 5.

The quantitative performance comparison on the Color Checker dataset is presented in Table 2 and the results on the NUS 8-Camera dataset in Table 3. The performance comparisons on the ADE20k (Zhou et al., 2017), SFU Lab dataset (Barnard et al., 2010), and SFU Gray-Ball dataset (200, 2003) are shown in Tables 4–6, respectively. Most CNN-based methods compare the effects on only two datasets, Color Checker dataset and NUS 8-Camera dataset. In order to make the comparison results consistent, the data in Tables 2, 3 are from Shi et al. (2016), while others were trained by us with the same training samples mentioned in the datasets section.

**Table 2:** Performance comparison on Color Checker dataset (Gehler et al., 2008).

**Method**	**Mean**	**Median**	**TriMean**	**Best 25%**	**Worst 25%**	**95th percentile**
*White-Patch (Brainard and Wandell, 1986)*	7.55	5.68	6.35	1.45	16.12	-
*Edge-based Gamut (Barnard, 2000)*	6.52	5.04	5.43	1.90	13.58	-
*Gray-World (Buchsbaum, 1980)*	6.36	6.28	6.28	2.33	10.58	11.3
*1st-order Gray-Edge (Weijer et al., 2007)*	5.33	4.52	4.73	1.86	10.03	11.0
*2nd-order Gray-Edge (Weijer et al., 2007)*	5.13	4.44	4.62	2.11	9.26	-
*Shades-of-Gray (Finlayson and Trezzi, 2004)*	4.93	4.01	4.23	1.14	10.20	11.9
*Bayesian (Gehler et al., 2008)*	4.82	3.46	3.88	1.26	10.49	-
*General Gray-World (Barnard et al., 2002)*	4.66	3.48	3.81	1.00	10.09	-
*Intersection-based Gamut (Gehler et al., 2008)*	4.20	2.39	2.93	0.51	10.70	-
*Pixel-based Gamut (Gehler et al., 2008)*	4.20	2.33	2.91	0.50	10.72	14.1
*Natural Image Statistics (?)*	4.19	3.13	3.45	1.00	9.22	11.7
*Bright Pixels (Joze et al., 2012)*	3.98	2.61	-	-	-	-
*Spatio-spectral (GenPrior) (Hirakawa et al., 2012)*	3.59	2.96	3.10	0.95	7.61	-
*Cheng et al. (2014)*	3.52	2.14	2.47	0.50	8.74	-
*Corrected-Moment (19 Color) (Finlayson, 2013)*	3.50	2.60	-	-	-	8.6
Corrected-Moment (19 Color)* (Finlayson, 2013)	2.96	2.15	2.37	0.64	6.69	-
*Corrected-Moment (19 Edge) (Finlayson, 2013)*	2.82	2.00	-	-	-	6.9
Corrected-Moment (19 Edge)* (Finlayson, 2013)	3.12	2.38	2.59	0.90	6.46	-
*Regression Tree (Cheng et al., 2015)*	2.42	1.65	1.75	0.38	5.87	-
*CNN (Bianco et al., 2015)*	2.36	1.98	-	-	-	-
*CCC (Barron, 2015)*	1.95	1.22	1.38	0.35	4.76	5.85
*DS-Net (Shi et al., 2016)*	1.90	1.12	1.33	0.31	4.84	5.99
*FC4 (Hu et al., 2017)*	1.77	1.11	1.29	0.34	4.29	5.44
*MSRWNS-AVG*	1.93	1.38	1.42	0.33	4.32	4.20
*MSRWNS-1*	1.72	1.16	1.33	0.32	3.79	4.36
*MSRWNS-2*	1.68	1.13	1.28	0.31	3.84	4.44
*MSRWNS-3*	1.71	1.13	1.31	0.31	3.82	4.18
*MSRWNS*	1.64	1.13	1.28	0.31	3.78	4.07

*For each metric, red indicates the best performance and blue indicates the second-best performance*.

**Table 3:** Performance comparison on NUS 8-Camera dataset (Cheng et al., 2014).

**Method**	**Mean**	**Median**	**TriMean**	**Best 25%**	**Worst 25%**
*White-Patch (Brainard and Wandell, 1986)*	10.62	10.58	10.49	1.86	19.45
*Edge-based Gamut (Barnard, 2000)*	8.43	7.05	7.37	2.41	16.08
*Pixel-Based Gamut (Gehler et al., 2008)*	7.70	6.71	6.90	2.51	14.05
*Intersection-based Gamut (Gehler et al., 2008)*	7.20	5.96	6.28	2.20	13.61
*Gray-World (Buchsbaum, 1980)*	4.14	3.20	3.39	0.90	9.00
*Bayesian (Gehler et al., 2008)*	3.67	2.73	2.91	0.82	8.21
*Natural Image Statistics (?)*	3.71	2.60	2.84	0.79	8.47
*Shades-of-Gray (Finlayson and Trezzi, 2004)*	3.40	2.57	2.73	0.77	7.41
*Spatio-spectral (ML) (Hirakawa et al., 2012)*	3.11	2.49	2.60	0.82	6.59
*2nd-order Gray-Edge (Weijer et al., 2007)*	3.20	2.26	2.44	0.75	7.27
*Bright Pixels (Joze et al., 2012)*	3.17	2.41	2.55	0.69	7.02
*1st-order Gray-Edge (Weijer et al., 2007)*	3.20	2.22	2.43	0.72	7.36
*Spatio-spectral (GenPrior) (Hirakawa et al., 2012)*	2.96	2.33	2.47	0.80	6.18
Corrected-Moment (19 Edge)*(Finlayson, 2013)	3.03	2.11	2.25	0.68	7.08
Corrected-Moment (19 Color)*(Finlayson, 2013)	3.05	1.90	2.13	0.65	7.41
*Cheng et al. (2014)*	2.96	2.04	2.24	0.62	6.61
*CCC (Barron, 2015)*	2.38	1.48	1.69	0.45	5.85
*Regression Tree (Cheng et al., 2015)*	2.36	1.59	1.74	0.49	5.54
*DS-Net (Shi et al., 2016)*	2.24	1.46	1.68	0.48	6.08
*FC4 (Hu et al., 2017)*	2.12	1.53	1.67	0.48	4.78
*MSRWNS-AVG*	2.13	1.51	1.72	0.58	5.44
*MSRWNS-1*	2.12	1.45	1.64	0.46	5.12
*MSRWNS-2*	2.11	1.45	1.66	0.51	5.29
*MSRWNS-3*	2.11	1.46	1.67	0.47	5.33
*MSRWNS*	2.11	1.45	1.64	0.45	4.77

*For each metric, red indicates the best performance and blue indicates the second-best performance*.

**Table 4:** Performance comparison on ADE20k dataset (Zhou et al., 2017).

**Method**	**Mean**	**Median**	**TriMean**	**Best 25%**	**Worst 25%**
*CCC (disc+ext) (Barron, 2015)*	2.14	1.66	1.82	0.32	4.24
*CNN (Bianco et al., 2015)*	1.96	1.32	1.14	0.23	3.94
*DS-Net (Shi et al., 2016)*	1.68	0.96	1.06	0.26	3.86
*FC4 (Hu et al., 2017)*	1.56	1.32	1.02	0.33	3.86
*MSRWNS-AVG*	1.66	0.96	1.44	0.32	3.66
*MSRWNS*	1.18	0.61	0.83	0.11	2.87

*For each metric, red indicates the best performance and blue indicates the second-best performance*.

**Table 5:** Performance comparison on SFU Lab dataset (Barnard et al., 2010).

**Method**	**Mean**	**Median**	**TriMean**	**Best 25%**	**Worst 25%**
*CCC (disc+ext) (Barron, 2015)*	3.77	2.19	2.21	0.42	8.87
*CNN (Bianco et al., 2015)*	3.18	2.31	2.40	0.39	6.98
*DS-Net (Shi et al., 2016)*	2.93	2.11	2.23	0.34	5.87
*FC4 (Hu et al., 2017)*	2.99	1.78	2.11	0.33	4.62
*MSRWNS-AVG*	3.15	1.88	2.01	0.32	4.88
*MSRWNS*	2.82	1.71	1.85	0.26	4.65

*For each metric, red indicates the best performance and blue the second-best performance*.

**Table 6:** Performance comparison on SFU Gray-Ball dataset (200, 2003).

**Method**	**Mean**	**Median**	**TriMean**	**Best 25%**	**Worst 25%**
*CCC (disc+ext) (Barron, 2015)*	3.31	1.66	1.82	0.32	4.24
*CNN (Bianco et al., 2015)*	2.96	1.32	1.14	0.23	3.94
*DS-Net (Shi et al., 2016)*	2.41	0.96	1.06	0.26	3.86
*FC4 (Hu et al., 2017)*	2.33	1.12	1.46	0.41	3.76
*MSRWNS-AVG*	2.18	1.12	1.34	0.26	3.88
*MSRWNS*	1.83	0.82	0.94	0.20	3.65

*For each metric, red indicates the best performance and blue indicates the second-best performance*.

**Figure 6:**
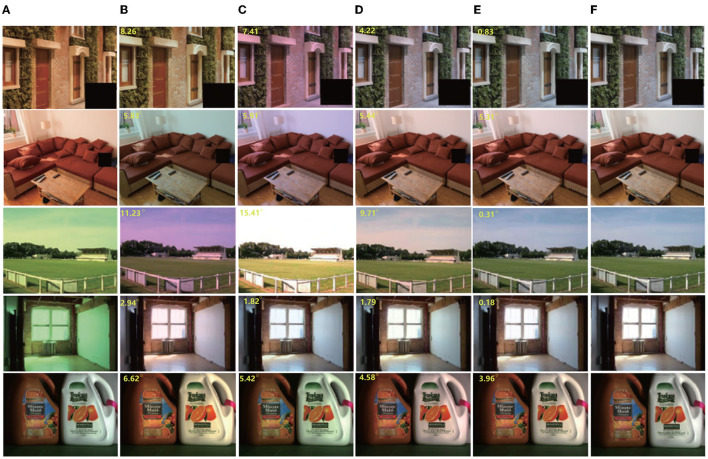
Visual comparison results. **(A)** Original image; **(B)** result obtained by CNN (Bianco et al., 2015); **(C)** result obtained by DS-Net (Shi et al., 2016); **(D)** result obtained by FC4 (Hu et al., 2017); **(E)** result obtained by proposed method; **(F)** ground truth. Regardless of quantification or visual effects, the proposed method shows better performance, especially in the first, second, and fourth lines, because there is a large area in the scene that can be accurately segmented, and the correction result of the method proposed in this article is very close to the real image. In the second line, because the color of the sofa and that of the light are relatively close. Although the network model considers the contribution of different regions, it is difficult to eliminate the color cast caused by the similar color of the light and the surface of the object. From the corrected image look, the image has a slight red tint. In the fourth line of the image, because the objects in the scene are too singular, the red objects on the left and the white objects on the right have greater contributions and the color of the objects on the left is biased in the result.

**Figure 7:**
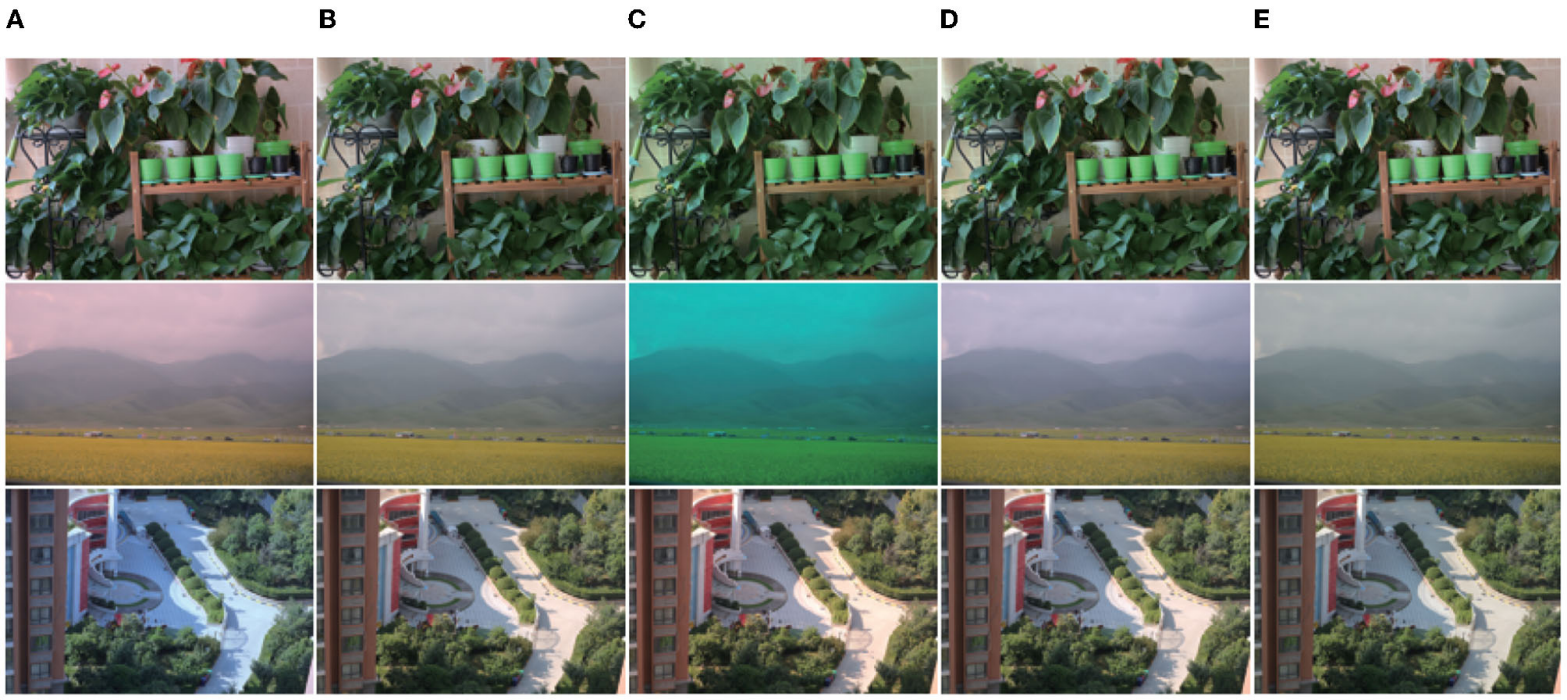
Visual comparison between the proposed method with the auto-white-balance (AWB) function of the Canon D7100 camera used. Left to right: **(A)** original image; **(B)** results by D7100 camera with AWB; **(C)** results by CNN; **(D)** results by DS-Net; **(E)** results by present study.

In addition, we compared our method with most of the existing works; typical works include the Deep Specialized Network (DS-Net) (Shi et al., 2016) and Fully Convolutional Color Constancy with confidence-weighted pooling (FC4) (Hu et al., 2017). Several visualizations of testing outputs obtained using the proposed method are presented in Figures 6, 7.

From Tables 2, 3, it can be seen that the mean error of the proposed method is reduced by 12.3% on the Color Checker dataset and by 5.8% compared to DS-Net (Shi et al., 2016), and reduced by 7.3% on the Color Checker dataset and reduced by 0.5% compared to FC4 (Hu et al., 2017). In addition, using the WPL layer under a single scale gives a better result than that obtained using the mean. The effect of using the mean of three scales on most indicators is better than the result of using a single scale.

In particular, the proposed method shows the best performance on the ADE20k (Zhou et al., 2017) dataset. The mean angular error is lower than that of DS-Net (Shi et al., 2016) by 29.7% (from 1.68 to 1.18), and the mean error of the worst 25% was reduced by 25.6% (from 3.86 to 2.87) compared to DS-Net (Shi et al., 2016). Other indicators have also been reduced to a certain extent. The reason for these results is that the semantic segmentation model used is trained based on the ADE20k (Zhou et al., 2017) dataset. The accuracy of the semantics on this dataset is high, and there are parts of training data in the ADE20k dataset (Zhou et al., 2017).

**Figure 8:**
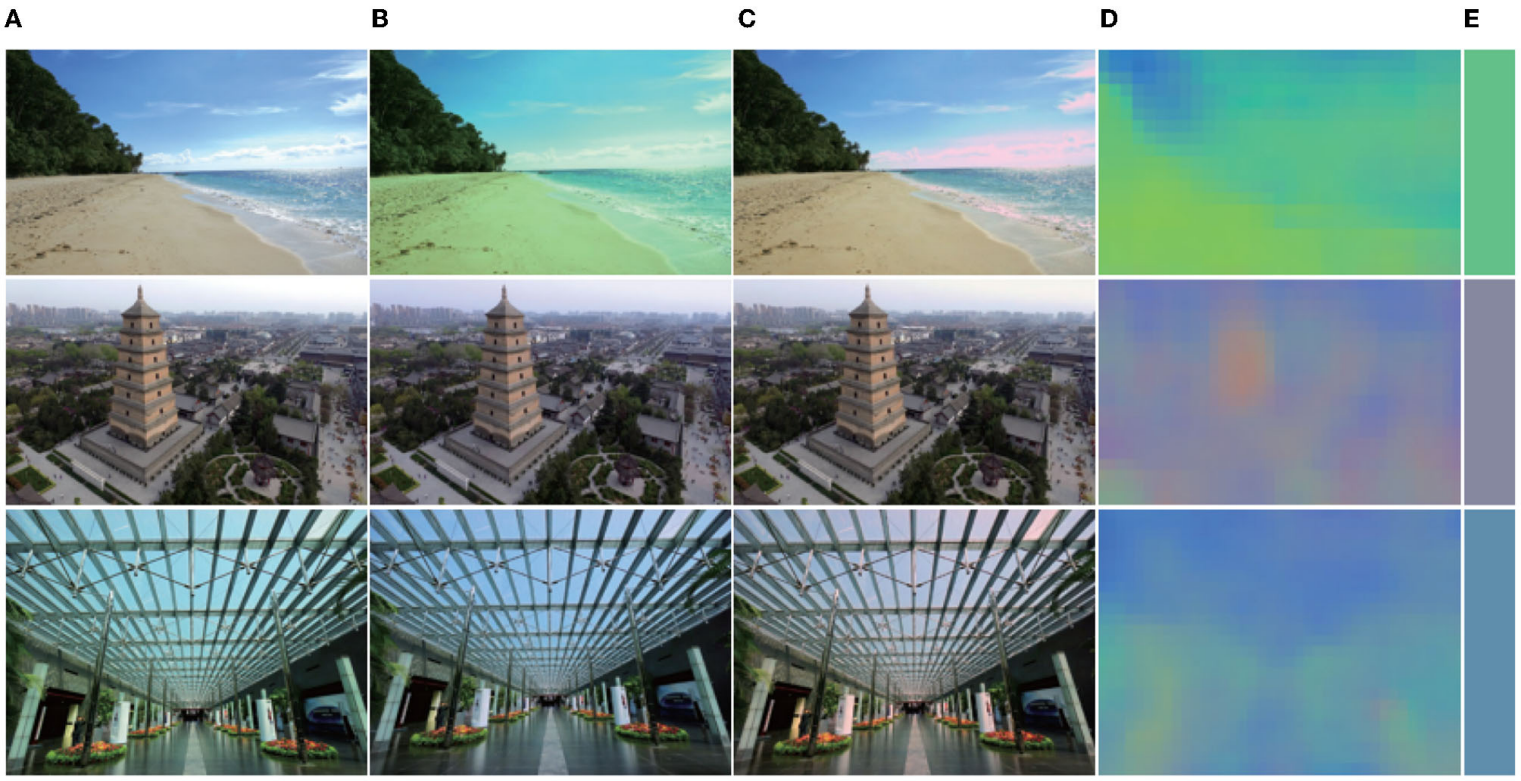
Result in a natural scene. From left to right. **(A)** Original image; **(B)** random color-cast image; **(C)** corrected image; **(D)** local-light image; **(E)** final-light color. It can be seen from the first row that, although there is a phenomenon of highlight overflow in some areas of the sky (due to random color casts leading to pixel overflow in some areas), the overall color is close to the original image; the second row is due to the random color cast. The resulting color cast is small, and the corrected image is basically the same as the original image. In the third row, it can be seen that, although the corrected image is different from the original image, the visual perception is more realistic.

In addition, we also provided several natural examples captured by a Canon D7100 without auto-white-balance ^5^, as shown in Figure 7, and we obtained several images of natural scenes with more accurate colors from the Internet, and then performed some random color casting. Results are shown in Figure 8.

It can be seen From Figure 7 that on the image taken indoors (the first line in Figure 7) the image corrected by a CNN (Bianco et al., 2015) is obviously yellowish, and that corrected by DS-Net is slightly greenish. The white-balance effect of the camera and our result is similar and look relatively natural. In outdoor scenes, it can be seen from the second line that the image corrected by a CNN (Bianco et al., 2015) has a very obvious color cast. The other types are visually more natural. Our results are seen in the sky part of the image, in which the clouds are more realistic. The scene in the third row exhibits little difference in visual effects. This may be due to sufficient sunlight in the shooting scene, and the objects in the scene receive very uniform illumination. In this way, any method can obtain better results more accurately. From Figure 8, it can be found from the first row that, although there is a phenomenon of highlight overflow in some areas of the sky (due to random color casts leading to pixel overflow in some areas), the overall color is close to that of the real image; the second row is due to the random color cast. The resulting color cast is small and the corrected image is basically the same as the original image. In the third row, it can be seen that, although the corrected image is different from the original image, the visual perception is better.

### 3.5. Efficiency

The code used to test the efficiency of the proposed method is based on Tensorflow (Rampasek and Goldenberg, 2016) and training took approximately 5 h, after which the loss tended to stabilize. In the testing phase, we converted the model to that of Caffe (Jia et al., 2014), implemented the WPL layer with C++ under Caffe, and finally used C++ code for testing. An average image took 34 ms on a CPU and only 12 ms on a GPU (the time does not include semantic segmentation) ^6^.

### 3.6. Adaptation for Multi-Illuminant

**Figure 9:**
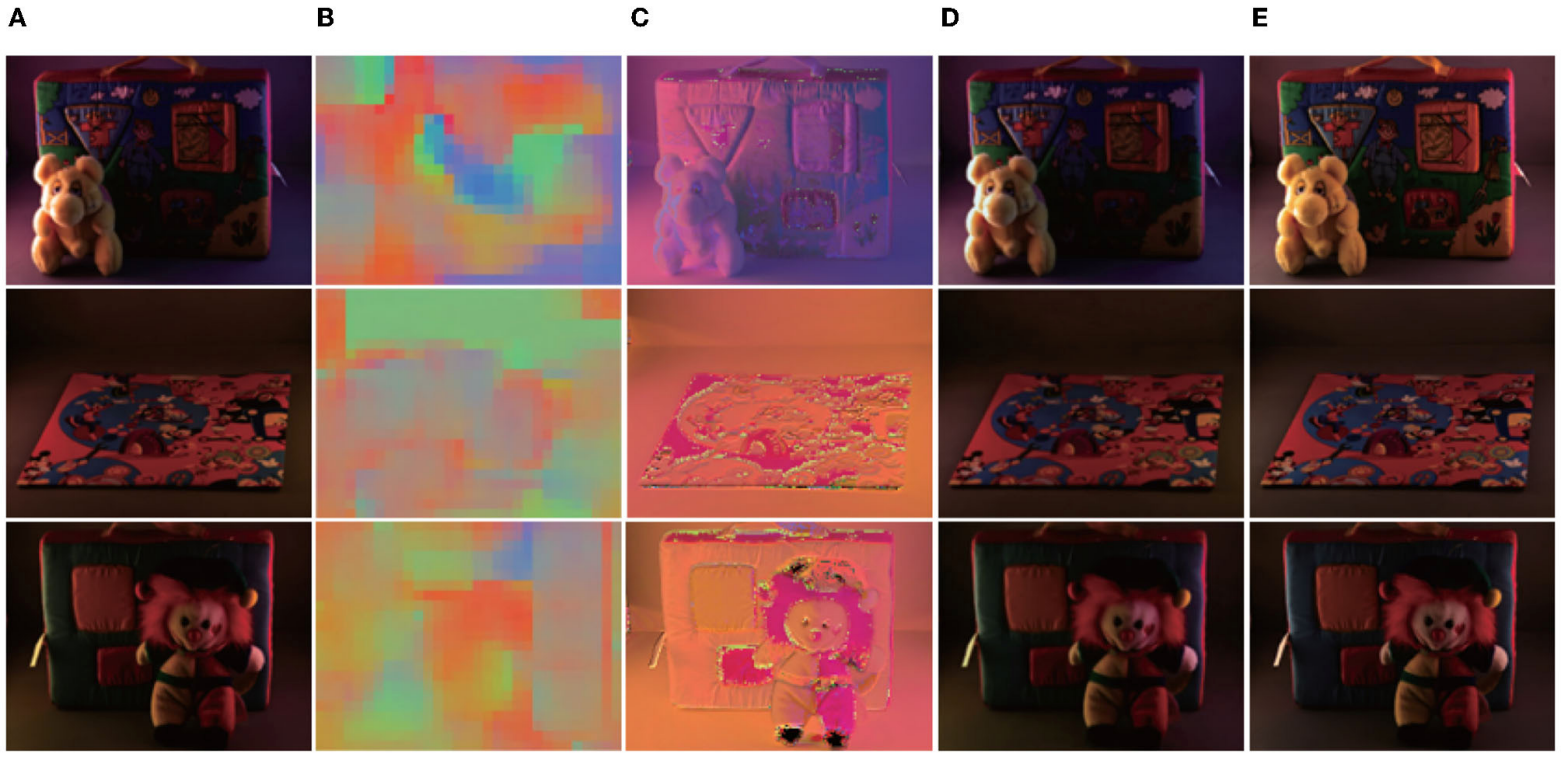
The evolution of illumination estimated. Left to right: **(A)** original image; **(B)** the estimated local illumination; **(C)** ground-truth illumination; **(D)** the corrected image by our method; **(E)** the ground-truth image.

As mentioned in this article, the proposed method aims to solve the color constancy under a single illuminant, and we only compare our algorithm with existing single illuminant based methods. In addition, after the WPL layer, we can get the local illumination of the regions, it can estimate the multi-illuminant sources in different local regions, shown in Figure 9, where the images are taken from the popular outdoor multi-illuminant dataset (Arjan et al., 2012). However, there has a large deviation between the estimated illumination and the real multi illumination, we have analyzed the reasons and found that there are big errors in semantics. We will solve this problem in future research.

## 4. Conclusion

In this article, we proposed a learning based multi-scale region weighed network guided by semantics (MSRWNS) to estimate the illuminated color of the light source in a scene. Cued by the human brain's processing of color constancy, we used image semantics and scale information to guide the process of illumination estimation. First, we put an image and its semantic mask into the network and, through a series of convolution layers, the region weights of the image at different scales were obtained. Then, through a WPL, the illumination estimation on each scale was obtained. Finally, we obtained the best estimation using the weighting on each scale, and state-of-the-art performance was achieved on three of the largest color-constancy datasets, i.e., the Color Checker, NUS 8-Camera, and ADE20k datasets. This study should prove applicable in the exploration of multi-scale and semantically directed networks for other fusion tasks in computer vision. In this study, we aim to solve the color-constancy problem with a single light source, however, there are multiple light sources in the real world, in our future research, we will try to solve the problem of multiple illuminations. In addition, it is time-consuming to obtain semantics, in our future work, we will try to use semantic information only in the training phase, not in the illumination estimation phase.

## Data Availability Statement

The original contributions presented in the study are included in the article/supplementary material, further inquiries can be directed to the corresponding author/s.

## Author Contributions

FW is responsible for conceptualization, investigation, data curation, and writing. WW is responsible for formal analysis, investigation, and methodology. DW is responsible for formal analysis, investigation, and validation. GG is responsible for data curation and investigation. All authors contributed to the article and approved the submitted version.

## Funding

This project was supported by the Science Fund of State Key Laboratory of Advanced Design and Manufacturing for Vehicle Body (No. 32015013).

## Conflict of Interest

The authors declare that the research was conducted in the absence of any commercial or financial relationships that could be construed as a potential conflict of interest.

## Publisher's Note

All claims expressed in this article are solely those of the authors and do not necessarily represent those of their affiliated organizations, or those of the publisher, the editors and the reviewers. Any product that may be evaluated in this article, or claim that may be made by its manufacturer, is not guaranteed or endorsed by the publisher.

## Footnotes

^1^In this study, we aim to solve the color-constancy problem with a single light source.

^2^As reported in Finlayson et al. (2004); Barron (2015), *log* − *uv* is better than RGB, since, first, there are two variables instead of three; and second, the multiplicative constraint of the illumination estimation model is converted to the linear constraint.

^3^In this work, the average value of multiple-scale illumination is used.

^4^We did not train the scene parsing network ourselves, the model used is downloaded from https://github.com/hszhao/PSPNet.

^5^The sensor of this camera does not have a low-pass filter, and the color filter comprises several Bayer filters that overlap each other.

^6^experimental hardware platform: i7 7700k, 32 GB memory, gtx1080ti. If the test-image resolution was greater than 512 × 512, it was resized to 512 × 512
